# Needle Arthroscopy as a Promising Alternative to MRI for the Diagnosis of Meniscus Injury

**DOI:** 10.7759/cureus.48671

**Published:** 2023-11-11

**Authors:** Tomoyuki Nakasa, Masakazu Ishikawa, Akinori Nekomoto, Kyohei Nakata, Takenori Omoto, Goki Kamei, Atsuo Nakamae, Nobuo Adachi

**Affiliations:** 1 Department of Artificial Joints and Biomaterials, Graduate School of Biomedical and Health Sciences, Hiroshima University, Hiroshima, JPN; 2 Department of Orthopaedic Surgery, Faculty of Medicine, Kagawa University, Takamatsu, JPN; 3 Department of Orthopaedic Surgery, Graduate School of Biomedical and Health Sciences, Hiroshima University, Hiroshima, JPN

**Keywords:** knee pain and functional impairment, specificity, sensitivity, diagnostic arthroscopy, mri, meniscus, needle arthroscopy

## Abstract

Meniscal injury is a common cause of knee pain and functional impairment, often necessitating surgical intervention. Although magnetic resonance imaging (MRI) is frequently used for diagnosis, its accuracy is variable and may lead to false positives and negatives. To address these issues, needle arthroscopy has gained attention as a potential diagnostic alternative to MRI because of its immediate availability and ability to directly visualize intra-articular structures. This study aimed to assess the diagnostic capabilities of needle arthroscopy in comparison with MRI and diagnostic arthroscopy for meniscal injuries. Forty patients with suspected meniscal injuries requiring surgical treatment were enrolled between November 2017 and March 2019. A needle arthroscope with a 0.95-mm diameter was used to evaluate meniscal injuries. Three orthopaedic surgeons with approximately 10 years of experience independently evaluated the images from the needle arthroscopy, diagnostic arthroscopy, and preoperative MRI without any knowledge regarding patients’ information. The sensitivity, specificity, positive predictive value (PPV), and negative predictive value (NPV) of each modality were used to compare the diagnostic accuracies. For lateral meniscus (LM) injuries, the sensitivity, specificity, PPV, and NPV of needle arthroscopy compared to diagnostic arthroscopy were 0.706, 0.852, 0.148, and 0.294, respectively. For medial meniscus (MM) injuries, the corresponding values were 0.889, 0.864, 0.136, and 0.111, respectively. In comparison, MRI had a lower sensitivity for LM injuries (0.588) and a higher sensitivity for MM injuries (1.0). The agreement between diagnostic arthroscopy and needle arthroscopy was moderate (kappa=0.517), while the agreements between diagnostic arthroscopy or needle arthroscopy and MRI were poor. Similar patterns were observed for the presence, location, and tear patterns of meniscal injuries. In conclusion, needle arthroscopy shows promise as an effective diagnostic modality for meniscal injuries, surpassing the limitations of MRI.

## Introduction

Meniscal injuries are commonly encountered in daily clinical settings and require treatment to prevent knee pain, functional impairment, and progression of osteoarthritis [1]. Magnetic resonance imaging (MRI) is routinely used in conjunction with physical examination to diagnose meniscal injury. However, MRI does not always accurately detect meniscal tears, and MRI findings often do not correlate with arthroscopic findings [2,3]. Moreover, diagnosis using MRI has high false-positive and false-negative rates, which may lead to unnecessary arthroscopic surgeries being performed due to false positives or delays in treatment due to false negatives [4,5]. The diagnostic accuracy of MRI can be affected by various factors, including magnetic strength and the physician’s level of experience [6]. As such, more accurate tools that are unaffected by such variable factors are required to diagnose meniscal injuries.

Although meniscal injury is usually diagnosed using MRI, arthroscopy is the gold standard for the final diagnosis [7]. However, the arthroscopic technique is expensive, invasive, and is associated with potential complications [8]. As such, the establishment of an accurate diagnosis before surgery is crucial. Recently, in-office needle arthroscopy has become widely used for the evaluation of intra-articular structures; this technique has been shown to have several strong advantages over MRI, including direct visualization and immediate availability in the office. Given these advantages, the use of diagnostic and therapeutic techniques using needle arthroscopy has increased [9-11], and this technique is expected to replace MRI as the new diagnostic modality. In addition, due to recent technological advances, needle arthroscopy currently has a diagnostic capability similar to surgical arthroscopy.

This study aimed to evaluate the diagnostic ability of needle arthroscopy for meniscal injuries through comparison with traditional MRI and diagnostic arthroscopy. We hypothesized that needle arthroscopy would have the same diagnostic ability for meniscal injury as diagnostic arthroscopy and a better diagnostic ability than MRI. 

## Materials and methods

Between November 2017 and March 2019, 40 patients were enrolled in this study. All patients were suspected to have a meniscal injury and underwent surgical treatment after conservative treatment failed to relieve their symptoms. They underwent preoperative MRI and arthroscopic surgery using conventional diagnostic arthroscopy and needle arthroscopy, and their images were retrospectively evaluated by three examiners. Only patients aged > 20 years who were preoperatively diagnosed with a meniscal injury were included. Severely obese patients with a BMI of 35 kg/m^2^ or greater were excluded. This study was approved by the Ethical Committee of Hiroshima University (approval number: C-119) and was registered in the Japan Registry of Clinical Trials (no. jRCTs062180102). Written informed consent was obtained from all participants enrolled in the study.

Needle arthroscopy system

**Figure 1:**
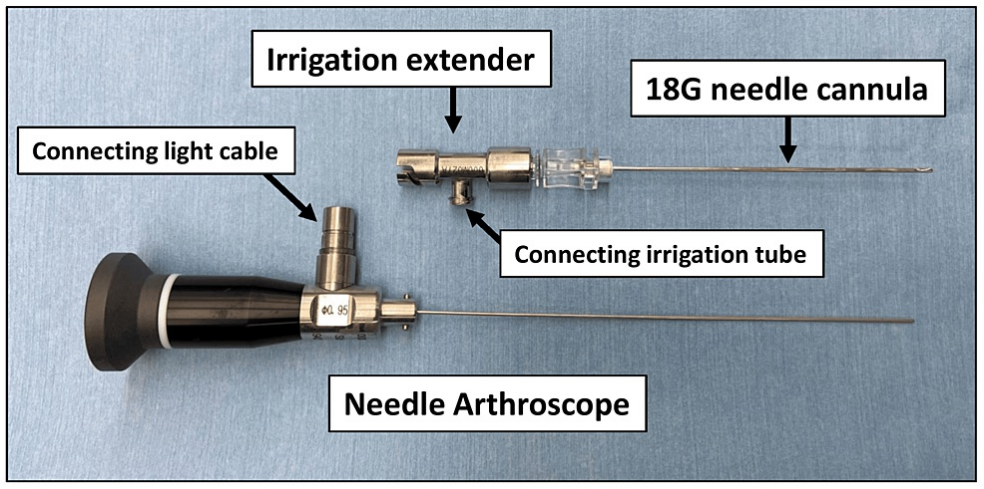
The needle arthroscope system. An 18G needle was used for the cannula of the arthroscope. An irrigation extender was connected to the irrigation system. The needle arthroscope was connected to the coupler and a light cable in a conventional arthroscope system and was sterilized in an autoclave before use. It could be used repeatedly.

The needle arthroscope (Smith & Nephew plc, London, United Kingdom) had a 0.95-mm diameter and a viewing angle of 115°. An 18G needle (diameter: 1.32 mm, length: 90 mm) was used for the cannula of the arthroscope, combined with an irrigation extender, which allowed clear vision by flushing the irrigation fluid through a syringe connected to the irrigation extender (Figure 1).

The needle arthroscope was connected to the coupler and a light cable in a conventional arthroscope system. It was sterilized in an autoclave before use and was used repeatedly like a conventional arthroscopy.

Arthroscopic examination

Arthroscopic surgery was performed under general anesthesia. After injecting approximately 80 cc of saline into the joint cavity, an 18 G cannula was inserted in the anterolateral portal, and the needle arthroscope was introduced into the cannula. When observing the medial compartment, the needle arthroscope was inserted in the anteromedial portal. The lateral and medial menisci were observed, and images were captured. Then, a conventional arthroscope was introduced in the knee joint to evaluate the diagnostic capability of the needle arthroscope. The cannulas were then removed, and 1 cm skin incisions were made as the anterolateral and medial portals. A standard arthroscope with a diameter of 4.5 mm and an irrigation system was introduced as the diagnostic arthroscope. The lateral and medial menisci were observed in the same manner as with the needle arthroscope and images were captured.

Evaluation

Three examiners with eight, nine, and 10 years of experience, respectively, as orthopaedic surgeons, who did not perform the arthroscopic surgery and know any patient information in this series, evaluated the surgical images of both the needle arthroscope and conventional diagnostic arthroscopy of the findings of menisci, and noted them on the sheet of the International of Society of Arthroscopy, Knee Surgery and Orthopaedic Sports Medicine (ISAKOS) classification of meniscal tears [12]. The examiners evaluated the meniscal findings on preoperative MRI according to the ISAKOS classification of meniscal tears. All images were reviewed in a randomized order, and patient information was blinded. The rates of agreement in the diagnosis of meniscal injuries between the needle arthroscope, conventional diagnostic arthroscopy, and MRI were compared.

Statistical analysis

The sensitivity, specificity, positive predictive value (PPV), and negative predictive value (NPV) for the diagnosis of meniscal injury using the needle arthroscope and MRI were calculated with reference to the results of conventional diagnostic arthroscopy. Comparisons of the findings obtained with needle arthroscopy, diagnostic arthroscopy, and MRI were performed using Cohen’s kappa coefficient (a measurement of agreement among the three readers). Kappa statistics were interpreted as follows: <0.40, poor agreement; 0.40 <kappa< 0.6, moderate agreement, 0.6 <kappa< 0.8, good agreement; >0.80, excellent agreement. Statistical analyses were performed using IBM SPSS Statistics for Windows, Version 22.0 (Released 2013; IBM Corp., Armonk, New York, United States), with p<0.05 set as statistically significant.

## Results

The cohort comprised 23 men and 17 women, with a mean age of 47.6 ± 15.6 years. The preoperative diagnoses were as follows: medial meniscal injury, 24 knees; lateral meniscal injury, 10 knees; and concurrent medial and lateral meniscal injuries, six knees. Diagnostic arthroscopy revealed that 31 of 40 (77.5%) patients had meniscal injuries and medial meniscal injury, 18 knees, lateral meniscal injury, 17 knees, and concurrent medial and lateral meniscal injuries, five knees. The sensitivity, specificity, PPV, and NPV of needle arthroscopy for the diagnosis of lateral meniscal injuries as compared with surgical diagnostic arthroscopy were 0.706, 0.852, 0.148, and 0.294, respectively. For medial meniscal injuries, these values were 0.889, 0.864, 0.136, and 0.111, respectively. In comparison, the corresponding values for the diagnostic ability of MRI compared with surgical diagnostic arthroscopy were 0.588, 0.818, 0.217, and 0.412, respectively for lateral meniscal injury, and 1.0, 0.591, 0.409, and 0, respectively, for medial meniscal injury.

Presence of meniscus injury

**Figure 2:**
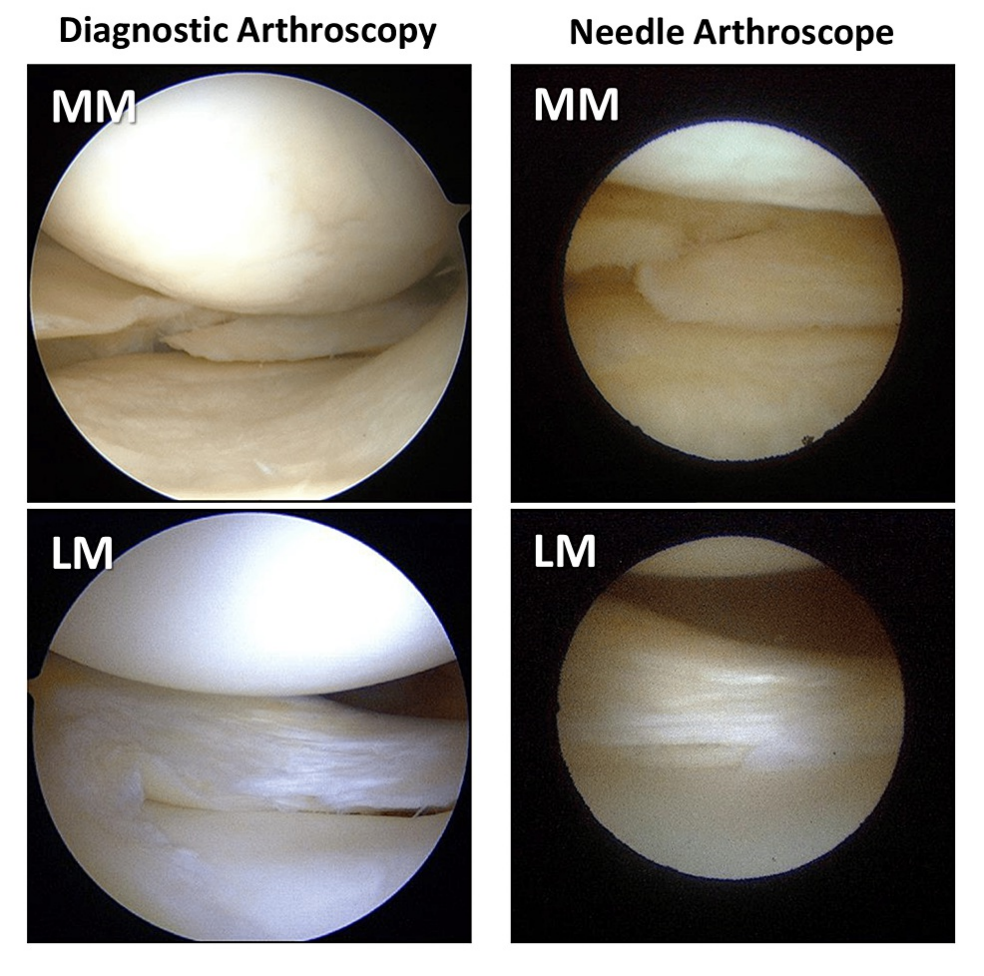
Findings of conventional diagnostic arthroscopy and needle arthroscopy for the lateral and medial menisci. The meniscal tears in both arthroscopic views were well-matched LM: lateral meniscus; MM: medial meniscus

**Figure 3:**
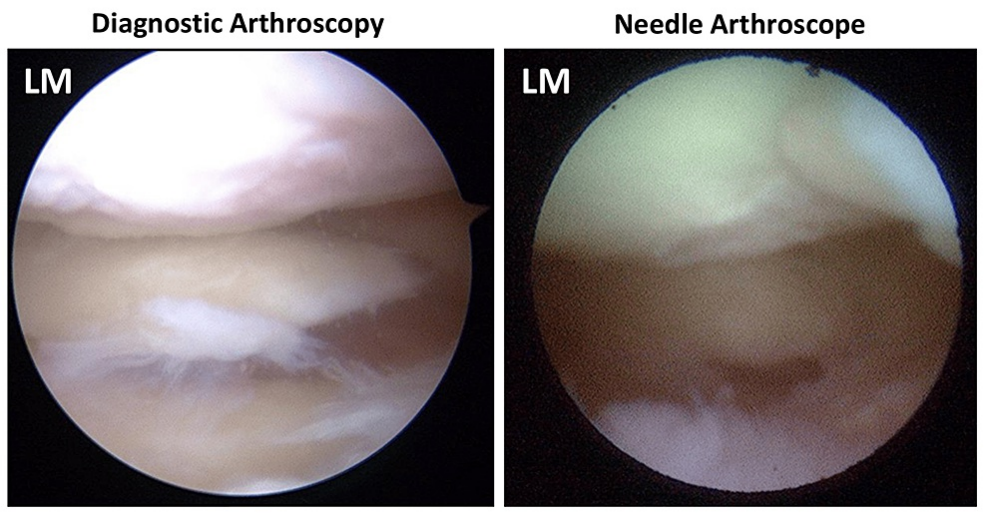
Representative case that could not be diagnosed with needle arthroscopy Lateral meniscal injury could be observed in conventional diagnostic arthroscopy but was not clearly visible in needle arthroscopy

For the comparison of diagnostic arthroscopy vs. needle arthroscopy, the kappa statistic was 0.517 (p<0.01), indicating a moderate agreement (Figures 2, 3).

In contrast, the comparisons between diagnostic arthroscopy and MRI, and needle arthroscopy and MRI, revealed poor agreements (kappa, 0.06 and 0.04, respectively) (Table 1). 

**Table 1:** Comparison between modalities regarding the presence of meniscal injury Diagnostic arthroscopy and needle arthroscopy indicated a moderate agreement, while diagnostic arthroscopy and MRI, and needle arthroscope and MRI, revealed poor agreements

	kappa	95%CI	p-value
Diagnostic arthroscope vs needle arthroscope	0.517	0.206, 0.829	<0.001
Diagnostic arthroscope vs MRI	0.061	-0.229, 0.351	0.64
Needle Arthroscope vs MRI	0.044	-0.225, 0.312	0.729

For the presence of medial meniscal injury, good agreement (kappa, 0.749; p<0.001) was observed between diagnostic arthroscopy and needle arthroscopy. Conversely, kappa statistics for diagnostic arthroscopy vs. MRI and needle arthroscopy vs. MRI were 0.565 (p<0.001) and 0.509 (p<0.001), respectively. For the presence of lateral meniscal injury, a moderate agreement between diagnostic arthroscopy and needle arthroscopy was observed (kappa, 0.637; p<0.001). However, the kappa statistics for diagnostic arthroscopy vs. MRI (kappa, 0.333, p<0.05) and needle arthroscopy vs. MRI (kappa, 0.123, p=0.433) were classified as poor (Table 2).

**Table 2:** Comparison between modality regarding the presence of lateral and medial meniscal injuries Good agreement was observed between diagnostic arthroscopy and needle arthroscopy for the presence of medial meniscal injury. A moderate agreement between diagnostic arthroscopy and needle arthroscopy was observed for lateral meniscal injury.

	Lateral meniscus			Medial meniscus		
	kappa	95%CI	p-value	kappa	95%CI	p-value
Diagnostic arthroscope vs needle arthroscope	0.637	0.402, 0.873	<0.001	0.749	0.543, 0.955	<0.001
Diagnostic arthroscope vs MRI	0.333	0.042, 0.625	0.033	0.565	0.34, 0.79	<0.001
Needle arthroscope vs MRI	0.123	-0.188, 0.435	0.433	0.509	0.266, 0.751	<0.001

Location of meniscus injury

The findings of needle arthroscopy, MRI, and diagnostic arthroscopy were consistent in 16 cases; the posterior segment of MM in 12 cases, the posterior segment of LM in three cases, and the middle segment of LM in one case. In eight cases, the findings of needle arthroscopy and conventional diagnostic arthroscopy were consistent; the posterior segment of LM in four cases, the posterior segment of MM in two cases, the anterior segment of LM in one case, and the middle segment of LM in one case. In three cases, the findings of the MRI and diagnostic arthroscopy were consistent; the posterior segment of MM in two cases, and the middle segment of LM in one case. For the location of the meniscal injury, good agreement (kappa, 0.613, p<0.001) was observed between diagnostic arthroscopy and needle arthroscopy. Conversely, kappa statistics for diagnostic arthroscopy vs. MRI and needle arthroscopy vs. MRI showed poor agreement (kappa 0.329; p<0.001, kappa 0.182; p=0.055, respectively) (Table 3).

**Table 3:** Comparison between modalities regarding the tear pattern of the meniscal injury For the diagnosis of the tear pattern of the meniscal injury, all comparisons showed poor agreement.

	kappa	95%CI	P value
Diagnostic arthroscope vs Needle Arthroscope	0.613	0.424, 0.802	<0.001
Diagnostic arthroscope vs MRI	0.329	0.131, 0.526	<0.001
Needle Arthroscope vs MRI	0.182	-0.001, 0.37	0.055

Tear pattern

For the diagnosis of the tear pattern of the meniscal injury, all comparisons (diagnostic arthroscopy vs. needle arthroscopy, diagnostic arthroscopy vs. MRI, and needle arthroscopy vs. MRI) showed poor agreement (Table 4). 

**Table 4:** Comparison between modalities regarding the tear pattern of the meniscal injury For the diagnosis of the tear pattern of the meniscal injury, all comparisons showed poor agreement.

	kappa	95%CI	p-value
Diagnostic arthroscope vs needle arthroscope	0.362	0.173, 0.552	<0.001
Diagnostic arthroscope vs MRI	0.215	0.055, 0.375	<0.001
Needle arthroscope vs MRI	0.137	-0.007, 0.28	0.029

## Discussion

This study demonstrated that needle arthroscopy showed good to moderate agreement with conventional diagnostic arthroscopy. Diagnostic arthroscopy is the gold standard for diagnosing meniscal injury; however, this surgical procedure must be performed in the operating room, making it invasive and costly for patients. In contrast, MRI is a non-invasive diagnostic method for meniscal injuries; however, its diagnostic accuracy is low. Regarding the diagnosis of lateral meniscal injury, the sensitivity and specificity of needle arthroscopy were higher than those of MRI. Conversely, in the diagnosis of medial meniscal injury, the sensitivity of MRI was higher than that of needle arthroscopy, but the specificity and false-PPVs were significantly higher for MRI. This suggests that MRI may lead to unnecessary surgery by diagnosing an uninjured medial meniscus as injured. Our study also revealed that the diagnostic ability for the tear pattern was lower than that for the presence and location of meniscal tears. The needle arthroscope has a narrower and darker field of view than diagnostic arthroscopy, which may make it more difficult to diagnose the tear patterns. However, it would be sufficient to diagnose the presence and location of the meniscal injuries in-office to determine the indication for surgical treatment. 

Recently, in-office needle arthroscopy has received increasing attention for its potential to change the therapeutic strategies for joint diseases and has been described as an alternative procedure to MRI for the diagnosis of intra-articular lesions. This technique has been reported to provide a potential solution to MRI-derived diagnostic inaccuracies, which can lead to missed lesions or incorrect treatment procedures [6]. Needle arthroscopy of the knee has been shown to have a 1.00 sensitivity for detecting chondral defects and 0.80 sensitivity for meniscal tears. Gill et al. further demonstrated that needle-based diagnostic imaging in the office was statistically equivalent to surgical diagnostic arthroscopy for the diagnosis of intra-articular, non-ligamentous knee joint pathology in a prospective, blinded, multicenter clinical trial [13]. Munn et al. also showed that the time to management was 45 days for needle scope versus 180 days for conventional arthroscopy, as needle arthroscopy can be implemented immediately at the office [14]. In-office needle arthroscopy has contributed significantly to the development of arthroscopic surgery. DiBartola et al. previously reported that healing of a meniscal tear repaired with a circumferential compression stitch was confirmed by in-office needle arthroscopy, which suggests that a second look can be performed in the office rather than the operating room [15]. Schaver et al. compared immediate post-operative pain and patient-reported outcomes following partial meniscectomy with needle versus standard arthroscopy techniques, finding that the adoption of a needle arthroscopic technique for partial meniscectomy was associated with significantly improved visual analog scale (VAS) and Knee injury and Osteoarthritis Outcome Score (KOOS) pain scores two weeks postoperatively [16].

The use of a needle scope can reduce the cost of the treatment of joint pathologies. Amin et al. demonstrated that the cost of diagnosing meniscal injury is lower with the needle scope than with MRI [17]. Moreover, the cost of surgery is also significant. Munn et al. demonstrated that outpatient needle arthroscopy can reduce costs compared with conventional knee arthroscopy [14]. Another report focusing on partial medial meniscectomy concluded that in-office needle arthroscopy is a cost-effective alternative to traditional arthroscopy in the operating room for patients undergoing partial medial meniscectomy, although the cost of the meniscal repair is unknown [18]. In addition, needle arthroscope produces two-thirds less non-recyclable waste than conventional arthroscopy, as fewer hospital resources were required [14]. We predict that, in the future, needle arthroscopy will be used not only for arthroscopic examinations in outpatient clinics, but also for a second look for postoperative follow-up, and preoperative arthroscopic examination for MRI-ineligible patients, as well as the observation of joint areas that are difficult to insert with conventional arthroscopy, and for use as a secondary scope in the operating room. Since the needle arthroscope has advantages that the risk of iatrogenic chondral damage by introducing the arthroscope can be reduced and it may allow observation of the posterior region of the knee without applying medial collateral ligament (MCL) crusting in tight knees, surgical procedures such as meniscal repair using a needle scope will develop in the future. With the needle scope widely available and advancing, we will have more options for diagnosing and treating meniscal injuries than ever before and will be able to choose a more individualized treatment strategy for each patient.

Several types of needle arthroscopes are commercially available. The needle arthroscope used in this study had a 0.95-mm diameter (the smallest available diameter), as well as a flexible scope. The needle arthroscope was developed to be inserted into an 18G needle, which is commonly used for joint puncture. In this arthroscope system, a clear view could be obtained by connecting a syringe to an irrigation extender device and performing flushing. Moreover, autoclave sterilization has been achieved for safe and low-cost operations. These features may facilitate the development of applications beyond office use. 

This study has several limitations which should be mentioned. First, the learning curve may have influenced the diagnosis by the needle arthroscopy, as the needle arthroscopy and diagnostic arthroscopy have different viewing angles. Second, the evaluation using needle arthroscopy was performed under general anesthesia because the diagnostic ability of the needle arthroscope was evaluated on the occasion of arthroscopic surgery under general anesthesia. As the diagnosis of the meniscal injury using the needle arthroscope in-office is assumed to be performed under local anesthesia, differences in pain control and muscle relaxation between general and local anesthesia may affect the visualization of the menisci. Finally, the three examiners had approximately 10 years of experience each as orthopaedic surgeons, which may have led to lower sensitivity and specificity for MRI and needle scope compared with other reports [6,13]. This limitation could have been improved by the inclusion of experts in knee surgery. However, as the diagnosis of meniscal injury using a needle arthroscope in the office becomes more popular, this procedure may end up being performed by surgeons who may not necessarily be knee experts. Therefore, it is significant that the needle scope findings matched the diagnostic arthroscope better than the MRI findings, even when evaluated by orthopaedic surgeons with approximately 10 years of experience.

## Conclusions

Needle arthroscopy demonstrated a promising diagnostic accuracy for meniscal injuries compared with MRI. As the popularity of this technique increases, it could become the preferred choice for diagnosing these injuries in clinical practice. This minimally invasive technique has the potential for use in the evaluation of meniscal injuries in the office, potentially reducing unnecessary arthroscopic surgeries and delays in treatment compared with traditional MRI. However, because MRI has the advantage of being able to evaluate a variety of extra-articular as well as intra-articular structures, a new algorithm, including the use of the needle arthroscope and MRI, should be developed in the future to make the therapeutic strategy for meniscal injury more effective.
